# Selective advantage of trisomic human cells cultured in non-standard conditions

**DOI:** 10.1038/srep22828

**Published:** 2016-03-09

**Authors:** Samuel D. Rutledge, Temple A. Douglas, Joshua M. Nicholson, Maria Vila-Casadesús, Courtney L. Kantzler, Darawalee Wangsa, Monika Barroso-Vilares, Shiv D. Kale, Elsa Logarinho, Daniela Cimini

**Affiliations:** 1 Department of Biological Sciences, Blacksburg, VA 24061 – USA; 2 Biocomplexity Institute, Virginia Tech, 1015 Life Sciences Circle, Blacksburg, VA 24061 – USA; 3 Biomedical Engineering, Virginia Tech, Blacksburg, VA 24061 – USA; 4 Bioinformatics Platform, CIBERehd, Barcelona, Spain; 5 Genetics Branch, National Cancer Institute, NIH, Bethesda, MD, 20892 – USA; 6 Aging and Aneuploidy Laboratory, Instituto de Biologia Molecular e Celular, Instituto de Investigação e Inovação em Saúde – i3S, Universidade do Porto, Rua Alfredo Allen 208, 4200-135 Porto – Portugal; 7 Cell Division Unit, Department of Experimental Biology, Faculdade de Medicina, Universidade do Porto, Alameda Prof. Hernâni Monteiro, 4200-319 Porto– Portugal

## Abstract

An abnormal chromosome number, a condition known as aneuploidy, is a ubiquitous feature of cancer cells. A number of studies have shown that aneuploidy impairs cellular fitness. However, there is also evidence that aneuploidy can arise in response to specific challenges and can confer a selective advantage under certain environmental stresses. Cancer cells are likely exposed to a number of challenging conditions arising within the tumor microenvironment. To investigate whether aneuploidy may confer a selective advantage to cancer cells, we employed a controlled experimental system. We used the diploid, colorectal cancer cell line DLD1 and two DLD1-derived cell lines carrying single-chromosome aneuploidies to assess a number of cancer cell properties. Such properties, which included rates of proliferation and apoptosis, anchorage-independent growth, and invasiveness, were assessed both under standard culture conditions and under conditions of stress (i.e., serum starvation, drug treatment, hypoxia). Similar experiments were performed in diploid vs. aneuploid non-transformed human primary cells. Overall, our data show that aneuploidy can confer selective advantage to human cells cultured under non-standard conditions. These findings indicate that aneuploidy can increase the adaptability of cells, even those, such as cancer cells, that are already characterized by increased proliferative capacity and aggressive tumorigenic phenotypes.

Fundamental to the survival of any organism is the balance between cell proliferation and cell death, which is required to ensure organismal development and to maintain healthy tissues and organs. The death and proliferation of normal, healthy cells is ensured by their ability to respond to and modulate growth and death signals. As opposed to healthy cells, cancer cells are characterized by the ability to escape such signals, thus becoming capable of evading apoptosis and proliferating independent of growth signals^1^. Several other features, typically referred to as “hallmarks of cancer”^1^, are shared by many cancer cells independent of their origin. One such feature, ubiquitous in cancer cells, is aneuploidy^2^,^3^,^4^. Inspired by his studies in sea urchin embryos, Theodor Boveri proposed, over a century ago, that the abnormal chromosome numbers (aneuploidy) found in cancer cells were responsible for cancer cells’ abnormal behavior^5^,^6^. Nevertheless, the effect of aneuploidy on cancer cell behavior is still unclear and abnormal chromosome numbers are generally acknowledged to negatively affect cell function^7^. Indeed, aneuploidy is the leading cause of miscarriage in humans^8^ and mosaic aneuploidy is typically associated with inherited disorders^9^. Moreover, recent studies aimed at investigating the effect of aneuploidy on cell physiology have revealed that aneuploidy negatively affects cellular fitness^7^ in a number of experimental systems, including mouse embryonic fibroblasts^10^ and budding yeast^11^. Nevertheless, there is also evidence that aneuploidy can confer a selective advantage in certain contexts. For instance, aneuploidy was shown to be an acquired trait in strains of *Candida albicans* that developed resistance to antifungal drugs^12^,^13^. Similarly, acquisition of aneuploid karyotypes was shown to allow budding yeast to adapt to a number of genotypic defects, including the lack of a key molecular motor^14^, telomerase insufficiency^15^, or lack of thiol peroxidase genes^16^. Moreover, aneuploid budding yeast strains were shown to display a growth advantage under a number of environmental stresses, despite their reduced fitness when grown under optimal conditions^17^. Finally, aneuploidy was proposed to contribute to the adaptation of liver cells in response to hepatic injury^18^,^19^ and is required for normal development of the Drosophila rectum^20^,^21^. These findings suggest that aneuploidy may confer a similar selective advantage to cancer cells. Moreover, the observation that certain aneuploidies can be either recurrent in cancers of different origin or specifically recurring in cancers from individual anatomical sites^22^ suggests that, as observed in fungi^12^,^13^,^17^ or in mouse hepatocytes^18^, specific aneuploidies may confer selective advantage in a given environment, but not in others.

Addressing the question of whether aneuploidy may confer a selective advantage to cancer cells can be very challenging, given that cancer cell karyotypes are very complex^2^,^22^,^23^ and typically characterized by high degrees of aneuploidy, as well as numerous chromosome rearrangements. Moreover, many cancer cells also display chromosome numerical instability (CIN), which generates chromosome numerical heterogeneity within cancer cell populations^3^,^24^,^25^. To avoid such complexity, we chose to address the effect of aneuploidy on cancer cells in a simplified experimental system. Specifically, we performed a series of assays in the diploid, chromosomally stable (non-CIN), colorectal cancer cell (CRC) line DLD1^24^ and two DLD1-derived cell lines that were previously generated via microcell-mediated chromosome transfer^26^ and carry an extra copy of either chromosome 7 (DLD1 + 7) or chromosome 13 (DLD1 + 13). Finally, we extended our investigation to primary human cells by performing cell proliferation experiments in diploid amniocytes (AF) and amniocytes with trisomy 13 (AF + 13). The trisomic cell lines used here (DLD1 + 7, DLD1 + 13, and AF + 13) were recently shown to display higher rates of whole-chromosome mis-segregation and to rapidly accumulate chromosome number heterogeneity compared to their diploid counterparts^27^.

## Results

To explore whether aneuploidy confers a selective advantage to cancer cells, we made use of two trisomic cell lines derived from the diploid (2N = 46), chromosomally stable CRC cell line DLD1^24^. The DLD1-derived trisomic cell lines used in this study carried an extra copy of either chromosome 7 (DLD1 + 7) or chromosome 13 (DLD1 + 13)^26^,^27^. This experimental set-up is advantageous for several reasons. First, it allows investigation of the specific effects of individual aneuploidies on the properties of cells that already display a transformed phenotype, thus providing insight on how aneuploidy may contribute to tumorigenesis. Second, it includes trisomies that may play different roles in cancer. Indeed, gain of chromosome 7 and chromosome 13 are both frequently found in colorectal cancer^28^,^29^. However, whereas gain of chromosome 7 is also seen in many other types of cancer^22^, gain of chromosome 13 appears to be specific to colorectal cancer^22^,^28^. We assessed a number of properties, including proliferative capacity, apoptosis evasion, anchorage-independent growth, and invasiveness, which are known to correlate with tumorigenic capacity^1^,^30^. Such properties were assessed under both standard culture conditions and under a number of culture conditions that are representative of possible conditions occurring in the tumor microenvironment^31^ concomitantly with the aneuploidies under study. We reasoned that this strategy would allow us to identify a potential selective advantage conferred by the extra chromosome. The selective conditions included nutrient starvation (serum-free culture medium), exposure to 10 μM of the chemotherapeutic drug 5-fluorouracil (5-FU), or hypoxia. The performance of the trisomic cells under such selective conditions was compared to that of the diploid parental CRC cell line.

### Aneuploidy suppresses cancer cell proliferation under standard culture conditions, but can confer a proliferative advantage under selective conditions

**Figure 1:**
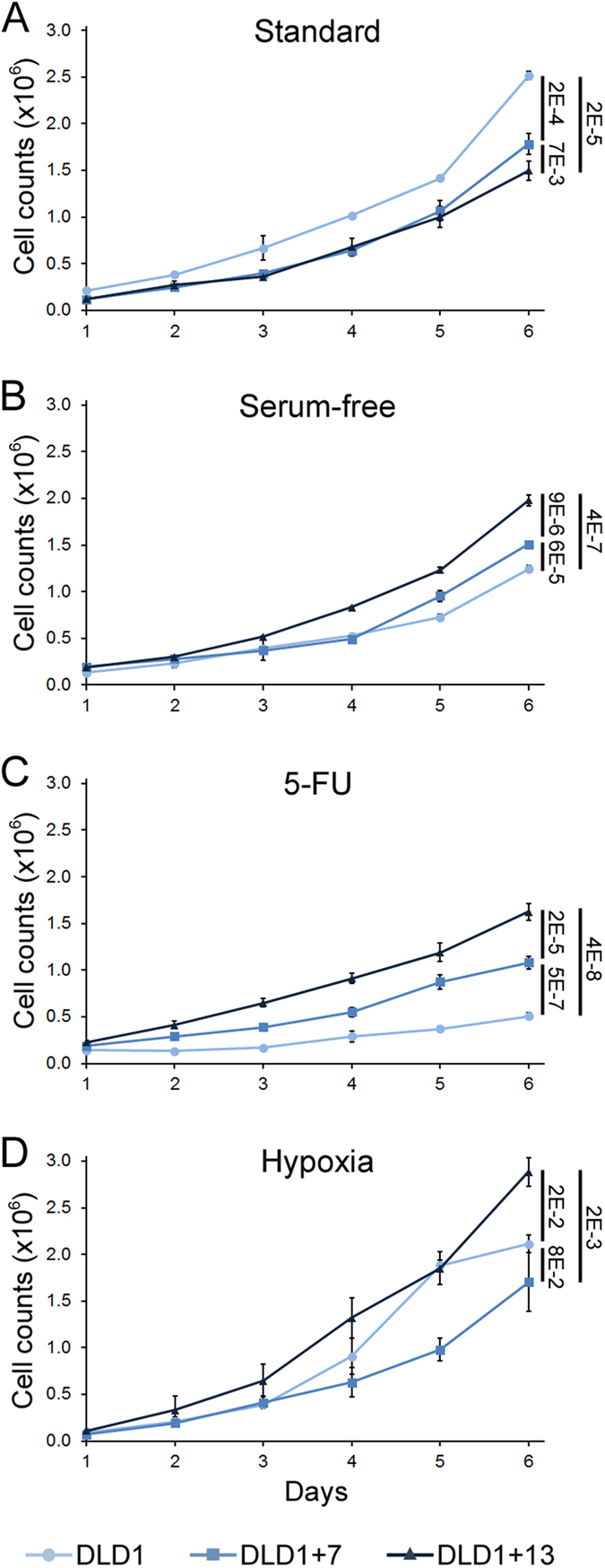
Aneuploidy suppresses cell proliferation under standard culture conditions, but favors proliferation under selective conditions in CRC cells. The graphs show growth curves for DLD1, DLD1 + 7, and DLD1 + 13 cells cultured for six days under different conditions. Data for cells cultured in standard media (**A**), serum-free media (**B**), media containing 10 μM 5-FU (**C**), or incubated under hypoxic conditions (**D**) are reported as mean and standard deviations from three biological replicates. Similar results were found by automated cell counting (data not shown). For statistical analysis, the growth curves in logarithmic scale were fit to linear functions and analyzed using R software package. The numbers to the right of the graphs indicate the p values for comparison of end-point data. Statistical analysis was also performed on the trends in cell growth using the geepack R package for longitudinal data analysis^60^ and showed statistically significant difference for all pairwise comparisons (p < 4.5E-7), except for DLD1 + 7 vs. DLD1 + 13 under standard culture conditions.

We first examined the proliferative capacity of the three CRC cell lines grown under various conditions by determining growth curves over a period of 6 days (Fig. 1). We found that under standard conditions, the DLD1 cells displayed faster growth rates compared to each aneuploid variant (Fig. 1A). Under both serum-free and 5-FU conditions (Fig. 1B,C), the proliferation rates were lower than those observed under standard culture conditions for all cell lines. However, the trisomic cell lines displayed higher proliferation rates than the parental diploid cell line, with the DLD1 + 13 cells proliferating better than both DLD1 + 7 and DLD1 cells (Fig. 1B,C). Moreover, the DLD1 + 13 cells displayed faster proliferation rates than either of the other two CRC cell lines, whereas the DLD1 + 7 cells did not display a proliferation advantage over the diploid cells under hypoxic conditions (Fig. 1D). However, it is important to note that the hypoxic conditions did not induce the overall reduction in proliferation rates observed under the other two selective conditions (compare Figs 1A–D). This may be explained by an adaptation of colorectal cells to survive in a hypoxic environment, such us the gut lumen^32^.

### Cell division and suppression of apoptosis in CRC cells cultured under standard and non-standard conditions

**Figure 2:**
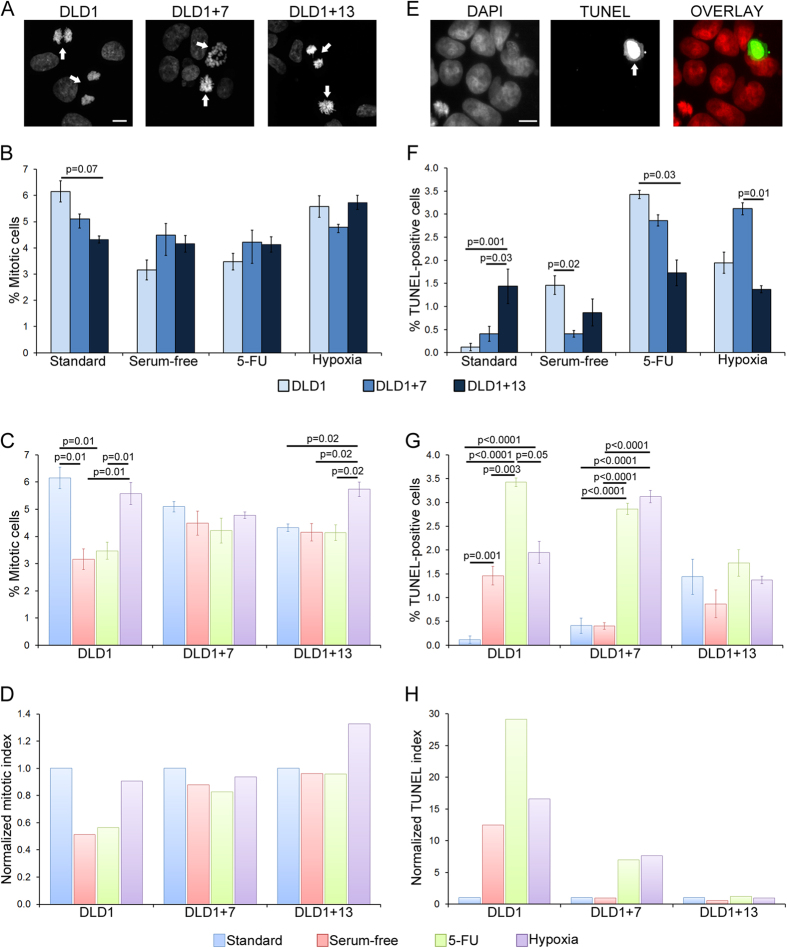
Mitotic index and apoptosis in diploid vs. aneuploid CRC cells. (**A**) Representative images of DLD1, DLD1 + 7, and DLD1 + 13 cells grown under standard culture conditions and stained with DAPI. Arrows point to mitotic cells. Images are maximum intensity projections of Z-stacks acquired at 0.6 μm steps. Scale bar, 10 μm. (**B,C**) Mitotic indices for the three cell lines cultured under different conditions and reported with data grouped either by culture conditions (**B**) or by cell line (**C**). The data are reported as mean and standard errors from three biological replicates. Statistical analysis was performed using the χ^2^ test and only p values that were significant or close to significance are reported in the figure. (**D**) The same data reported in (**C**) are shown here as normalized value over the mitotic index of the given cell line cultured under standard conditions. (**E**) Representative TUNEL assay image of DLD1 + 13 cells cultured under standard conditions. Arrow points to TUNEL-positive cell. Images are maximum intensity projections of Z-stacks acquired at 0.6 μm steps. Scale bar, 10 μm. (**F,G**) Quantification of TUNEL-positive cells in the three cell lines cultured under different conditions and reported with data grouped either by culture conditions (**F**) or by cell line (**G**). The data are reported as mean and standard errors from three biological replicates. Statistical analysis was performed using the χ^2^ test and only p values that were significant or close to significance are reported in the figure. (**H**) The same data reported in (**G**) are shown here as normalized value over the TUNEL-positive values of the given cell line cultured under standard conditions.

To assess whether the higher proliferation rates were linked to increased cell division rates, we quantified the mitotic index for the three CRC cell lines under all culture conditions (Fig. 2A–D). We found that the diploid cells displayed a higher mitotic index compared to the aneuploid CRC cells under standard culture conditions (Fig. 2B), whereas both trisomic CRC cell lines displayed slightly higher (but not statistically significant) mitotic indices compared to DLD1 cells grown under serum-free or 5-FU conditions (Fig. 2B). Under hypoxic conditions, the DLD1 + 7 cells displayed the lowest mitotic index (although the difference was not statistically significant), whereas DLD1 and DLD1 + 13 cells displayed similar mitotic indices (Fig. 2B). However, it is worth noting that the mitotic indices displayed by all cell lines under hypoxic conditions were higher (statistically significant for both DLD1 and DLD1 + 13) than those observed under either of the other two selective conditions and similar to those observed under standard culture conditions (Fig. 2C,D). More strikingly, the mitotic index for DLD1 + 13 cells subjected to hypoxia was higher than the mitotic index measured for DLD1 + 13 cells under any other culture condition (Fig. 2C,D), consistent with the high proliferation rate of these cells in hypoxia (Fig. 1D) and suggesting a karyotype-specific effect of trisomy 13 in adaptation to hypoxia in colorectal cancer.

Because overall proliferation rates are determined by the combined rates of cell division and cell death, and because cancer cells are known to evade apoptosis^1^, we also quantified the rates of apoptosis by TUNEL assay in the three CRC cell lines under all culture conditions (Fig. 2E–H). Under standard culture conditions, DLD1 cells showed the lowest incidence of apoptosis, whereas their rate of apoptosis was the highest among the three cell lines both under serum-free and 5-FU conditions (Fig. 2F). Under hypoxic conditions, the DLD1 + 7 cells displayed the highest rate of apoptosis among the three CRC cell lines (Fig. 2F), which is consistent with the observation that DLD1 + 7 cells displayed the lowest proliferation rates among the three cell lines under hypoxic conditions (Fig. 1D). The TUNEL assay data also showed that the rates of apoptosis did not vary significantly for DLD1 + 13 cells grown under different conditions (Fig. 2G,H), whereas the DLD1 cells showed higher rates of apoptosis under all selective conditions compared to standard culture conditions (Fig. 2G,H), and DLD1 + 7 cells displayed lower rates of apoptosis under standard and serum-free conditions compared to their rates of apoptosis in 5-FU and hypoxia (Fig. 2G,H).

To determine whether the observed mitotic indices and rates of apoptosis could explain the growth trends of the three CRC cell lines cultured under different conditions, we used a deterministic mathematical model (supplementary information) that took into account the experimentally observed mitotic indices and rates of apoptosis (Fig. 2) as well as mitotic timing (Table S1) for the three CRC cell lines. Simulation data obtained using this model showed that the experimentally observed growth curves indicated slower proliferation than what could be accounted for based on mitotic indices and rates of apoptotic death, suggesting that cell death may occur in our CRC cell lines at rates higher than those detected by TUNEL assay (see supplementary information and data therein for further information and discussion).

### Aneuploidy can increase anchorage-independent growth of CRC cells

**Figure 3:**
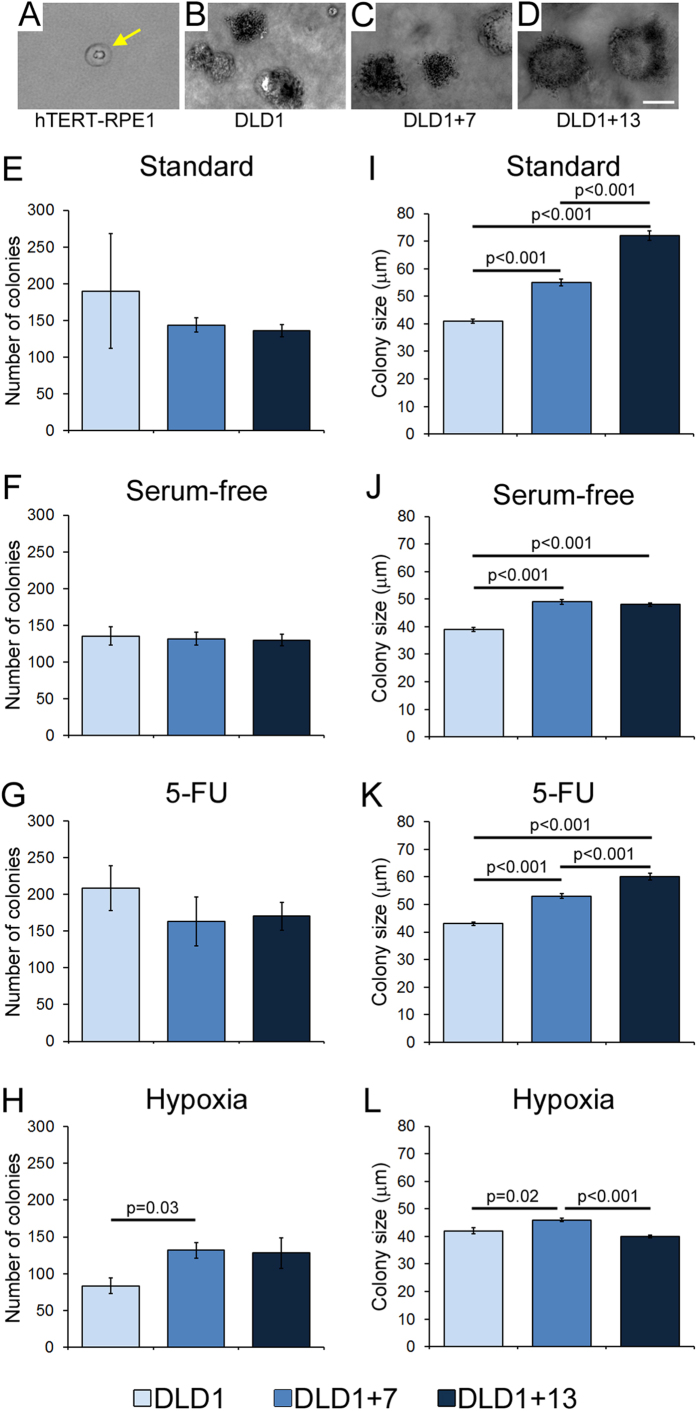
Aneuploidy can increase anchorage-independent growth in CRC cells. Anchorage-independent growth was assessed by testing the ability of DLD1, DLD1 + 7, and DLD1 + 13 cells to form colonies on soft agar when cultured under different conditions. (**A**) Representative image of hTERT-RPE1 cells on soft agar. These non-transformed cells were used as a negative control and as a baseline for colony size measurements. Individual dot-like structures (yellow arrow), corresponding to individual non-proliferating cells, were observed at low density. The average size of these structures was 15 μm, and this was considered as the lower size limit for colony size measurements in DLD1 cell lines, although most of the cancer cell line colonies were well above 15 μm in size. (**B–D**) Representative colonies formed by DLD1, DLD1 + 7, and DLD1 + 13 on soft agar under standard culture conditions. Scale bar, 50 μm. (**E–H**) Total number of colonies from ten randomly selected fields of view of soft agar plates with the various cell lines under different culture conditions. (**I–L**) Average size of colonies formed by the various cell lines under different culture conditions. For (**E–L**), the data are reported as mean and standard errors from three biological replicates. Statistical analysis was performed using the t-test and only p values that were significant or close to significance are reported in the figure.

We next performed a soft agar assay to examine the ability of the three CRC cell lines to grow independent of anchorage *in vitro*, a phenotype that correlates with tumorigenicity^30^. As a negative control we used the non-transformed, immortalized, hTERT-RPE1 cell line. As expected, these cells were unable to form colonies in soft agar (Fig. 3A). On the other hand, all three DLD1 cell lines formed colonies under all culture conditions (Fig. 3B–H), consistent with anchorage-independent growth being the phenotype most consistently associated with tumorigenicity^30^. The three CRC cell lines formed similar numbers of colonies under most culture conditions (Fig. 3E–H), with the exception of hypoxia, in which DLD1 + 7 CRC cells formed more colonies compared to the diploid DLD1 cells (Fig. 3H). Moreover, in most cases the colonies formed by the aneuploid CRC cells were significantly larger than those formed by their diploid counterparts (Fig. 3I–L). Notably, the aneuploid CRC cell lines also formed larger colonies compared to diploid cells when cultured under standard conditions (Fig. 3I).

### Aneuploidy increases the invasive capacity of CRC cells

**Figure 4:**
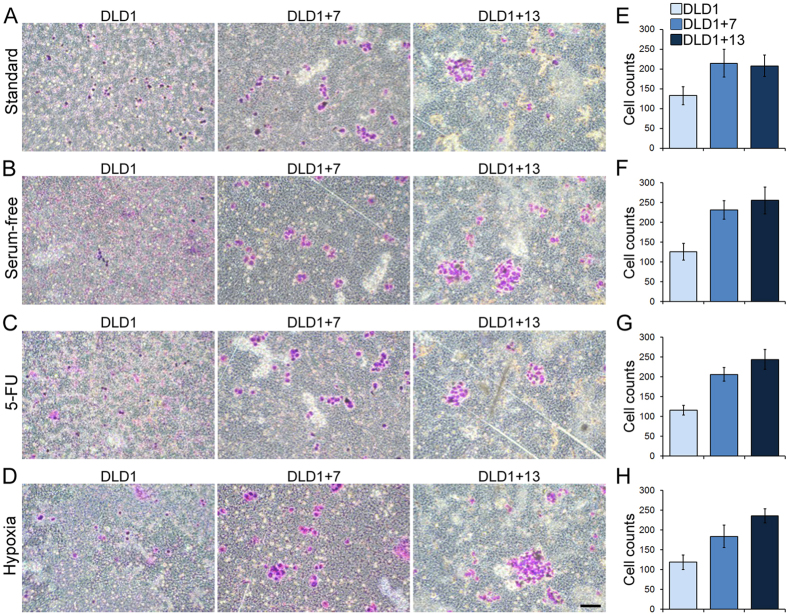
Aneuploidy increases invasiveness of CRC cells. The invasive capacity of the three different cell lines was assessed using a matrigel invasion assay. (**A–D**) Examples of Giemsa-stained invasive DLD1, DLD1 + 7, and DLD1 + 13 cells cultured under different conditions. Scale bar, 100 μm. (**E–H**) Quantification of invasive DLD1, DLD1 + 7, and DLD1 + 13 cells cultured under different conditions. The data are reported as mean and s.e.m. from three biological replicates. Statistical analysis showed that significantly larger numbers of aneuploid compared to diploid cells migrated through the matrigel layer (t-test, p < 10^−4^ for each aneuploid CRC cell line compared to diploid cells under all culture conditions).

To assess the invasive ability of aneuploid CRC cells compared to the diploid parental cell line, we performed a matrigel invasion assay (Fig. 4). We found that under all culture conditions, the number of cells migrating through the matrigel was higher for the aneuploid CRC cell lines compared to the diploid parental cell line (t-test, p < 10^−4^ for each aneuploid cell line compared to diploid cells under all culture conditions; Fig. 4E–H).

### Trisomy 13 confers a proliferative advantage to primary human cells cultured under selective conditions

**Figure 5:**
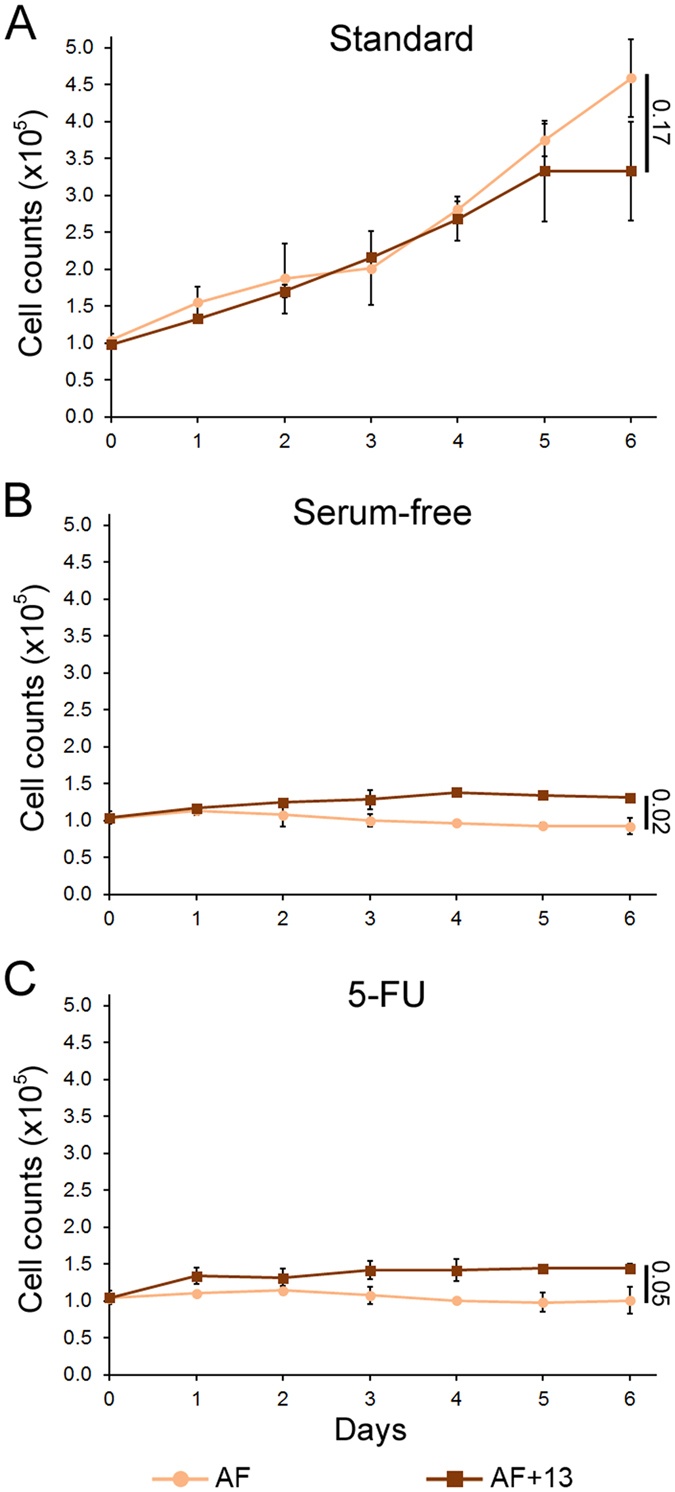
Aneuploidy favors proliferation of non-transformed amniotic fibroblasts grown under non-standard conditions. The graphs show growth curves for AF and AF + 13 cells cultured for six days under different conditions (treatment starting at day 0). Data for cells cultured in standard media (**A**), serum-free media (**B**), or media containing 5 μM 5-FU (**C**) are reported as mean and standard deviations from 2–3 biological replicates. For statistical analysis, the growth curves in logarithmic scale were fit to linear functions and analyzed using R software package. The numbers to the right of the graphs indicate the p values for comparison of end-point data. Statistical analysis was also performed on the trends in cell growth using the geepack R package for longitudinal data analysis^60^ and showed statistically significant difference for all pairwise comparisons (Standard, p = 0.02; Serum-free and 5-FU, p < 1E-6).

We next assessed the ability of aneuploidy to confer a selective advantage to non-transformed human cells by using diploid amniocytes (AF) and amniocytes with trisomy 13 (AF + 13). The proliferative capacity of these cells was assessed under standard culture conditions, in serum-free media, or in the presence of 5 μM 5-FU. We found that under standard culture conditions the diploid cells proliferated slightly better than the AF + 13 cells (Fig. 5A). However, we found that under both selective conditions, proliferation of the diploid cells was completely abrogated, whereas the aneuploid amniocytes were still capable of proliferating (Fig. 5B,C). Although the rates of proliferation of AF + 13 cells was dramatically reduced under selective conditions compared to their proliferation under standard culture conditions, the rates of proliferation of AF + 13 cells were significantly higher than their diploid counterparts cultured under the same selective conditions (Fig. 5B,C).

## Discussion

Taken together, our data show that aneuploidy can confer a selective advantage to human cells under conditions of environmental stress, although in some of our assays and under certain culture conditions, there was no difference in behavior between diploid and aneuploid CRC cells. For both the CRC cells and the amniocytes, the diploid cells proliferated better under standard culture conditions. A similar proliferative advantage of euploid vs. aneuploid cells under standard culture conditions was previously shown in both yeast and mouse embryonic fibroblasts^10^,^11^,^17^,^33^. On the other hand, we found that the aneuploid cells displayed a proliferative advantage under selective culture conditions, indicating that aneuploidy can confer a selective advantage to cancer cells, as previously shown in yeast^14^,^17^,^33^. Our results are also consistent with the previous finding that trisomy 7 was associated with an acquired resistance of colon epithelial cells to growth in serum-free conditions^34^. However, our findings are even more striking in that they show that aneuploidy increases the tolerance of CRC cells to environmental stresses beyond that seen in cells that are already transformed and tumorigenic, and presumably already adapted to proliferate under certain environmental stresses.

Aneuploidy has been shown to cause transcriptomic changes that, for the most part, correlate with the specific aneuploid chromosome(s)^11^,^17^,^26^,^35^,^36^,^37^. However, when examining the proteomic changes linked to aneuploidy, the situation is more complex. Whereas some studies have reported proteomic changes to scale up with changes in chromosome copy number in yeast^17^, other studies in yeast and human cells reported that protein abundance does not vary significantly as a result of aneuploidy and/or that the proteomic changes do not necessarily correlate with the specific aneuploidy^11^,^36^,^37^,^38^. Nonetheless, a general link between aneuploidy and phenotypic traits is strongly supported by experimental data^17^,^39^,^40^,^41^. Moreover, aneuploidy-specific phenotypes, resulting from the altered levels of proteins encoded by genes on the aneuploid chromosomes, have been reported in a number of different organisms and contexts. For instance, the chromosome 5 aneuploidy emerging in *Candida albicans* exposed to fluconazole^12^,^13^ was shown to confer drug resistance by inducing overexpression of two genes encoding, respectively, for the drug target and for a transcriptional regulator of efflux pumps^42^. Similarly, the acquisition of trisomy 7 in colon epithelial cells cultured in serum-free conditions was associated with overexpression of the epidermal growth factor receptor, encoded on chromosome 7^39^. Finally, a specific cytokinesis failure phenotype was recently shown to be associated with trisomy 13 and to be caused by overexpression of Spartin, a protein encoded by a gene (SPG20) on chromosome 13^27^. The selective advantage we observed in our study seems unlikely to depend on overexpression of specific genes on the aneuploid chromosomes. If that were the case, we would expect to find consistently significant differences between trisomy 7 and trisomy 13. In other words, we would expect trisomy 7 to confer selective advantage under a given culture condition and trisomy 13 to confer selective advantage under a different culture condition, which was not the case in our experiments. Therefore, although the specificity of chromosome 13 gain in colorectal cancer^22^ may suggest a specific advantage conferred by this chromosome to CRC cells, such advantage may be linked to conditions other than those tested in our study. Instead, for the selective conditions assessed here, we found a general selective advantage conferred by aneuploidy. This observation suggests that the advantage conferred by these two chromosomes may depend on a common phenotypic effect observed for both trisomies. Such an effect may be the increased rate of chromosome mis-segregation recently reported for DLD1 + 7, DLD1 + 13, and AF + 13 cells compared to diploid DLD1 and AF cells^27^. In DLD1 + 7, DLD1 + 13, and AF + 13 cells, high rates of chromosome mis-segregation are associated with high rates of karyotypic heterogeneity, which become apparent in the population even when cells are examined at relatively low passages^27^; this is consistent with findings from other studies showing a link between aneuploidy and chromosome instability (CIN) in both yeast and human cells^39^,^41^,^43^,^44^,^45^,^46^. Cell populations with high rates of karyotypic heterogeneity are expected to display high degrees of phenotypic heterogeneity^33^,^47^ and this karyotypic/phenotypic heterogeneity will allow aneuploid cells to be more “adaptable”^33^,^47^. Indeed, high karyotypic/phenotypic heterogeneity increases the chance that some cells within the population may possess the phenotype required for increased fitness in a certain environment.

Recent studies have shown that despite a general correlation between genes on the aneuploid chromosome and gene expression/proteomic profiles^10^,^17^,^35^,^36^,^48^,^49^,^50^, upregulation of transcripts and/or proteins involved in stress response can occur independent of the specific aneuploidy^36^,^49^,^50^,^51^. This observation suggests that the upregulation of stress response pathways may make aneuploid cells “primed” for a response to environmental changes, thus contributing (alone or in concert with the karyotypic/phenotypic heterogeneity arising in aneuploid cell populations) to confer a selective advantage to aneuploid cells.

Our soft agar and matrigel invasion assays provided interesting observations in that aneuploid CRC cells were found to grow larger colonies and to be more invasive than diploid CRC cells not only under selective conditions, but also under standard culture conditions. One could envision anchorage-independent growth or migration through an extra-cellular matrix as challenges of their own. Therefore, the observation that aneuploid cells are more anchorage-independent and migrate better through matrigel regardless of the culture conditions, reinforces the idea that aneuploidy increases the fitness of cells, even those, such as cancer cells, that are already characterized by increased proliferative capacity and aggressive tumorigenic phenotypes.

In several of our assays, DLD1 + 13 cells performed better than DLD1 + 7 cells, despite both displaying an advantage over DLD1 cells. DLD1 + 13 cells were recently shown to fail cytokinesis and acquire tetraploid/near-tetraploid karyotypes at significantly higher rates compared to the other two CRC cell lines^27^. Interestingly, tetraploidy was recently shown to increase the tolerance for mitotic errors and CIN^52^,^53^. Thus, the better fitness of DLD1 + 13 over DLD1 + 7 may be explained by the ability of tetraploidy to buffer the overall effects of aneuploidy and confer tolerance to phenomena/events other than mitotic errors and CIN.

Given the advantage provided to cancer cells by aneuploidy, it is surprising that non-CIN CRC cells (such as DLD1) exist. However, this may be due to the fact that a combination of the high rates of mutation displayed by non-CIN CRC cells^54^ and the increase in CIN triggered by aneuploidy may not be tolerated and may cause cell death rather than a proliferative advantage. This is consistent with the previously suggested tumor-suppressing effect of very high rates of CIN^55^.

In conclusion, our work shows that addition of one extra chromosome is enough to confer a selective advantage to both primary and cancer cells, allowing aneuploid cells to perform better than diploid cells under a number of environmental challenges. These findings are consistent with the widespread aneuploidy observed in solid tumors^4^, with the association between aneuploidy/CIN and poor patient prognosis^56^,^57^, and with the link between CIN and drug resistance^58^,^59^.

## Materials and Methods

### CRC and hTERT-RPE1 cell lines: general information, culture conditions, and treatments

The DLD1 cell line was obtained from American Type Culture Collection (ATCC, VA, USA); the DLD1 + 7 and DLD1 + 13 cell lines were generated previously by microcell-mediated chromosome transfer^26^, and DLD1 + 13 cells were sub-cloned as previously described^27^. All the DLD1 cell lines were maintained in RPMI 1640 medium (ATCC, VA, USA) supplemented with 10% FBS (Gibco, NY, USA) and antibiotic/antimycotic mixture (Gibco, NY, USA). The hTERT-RPE1 cells (ATCC, VA, USA) were maintained in DMEM/F12 medium (Gibco, NY, USA) supplemented with 10% FBS (Gibco, NY, USA) and antibiotic/antimycotic mixture (Gibco, NY, USA). All cells were kept in a humidified incubator at 37 °C with 5% CO_2_.

For exposure to selective conditions, culture medium was prepared as follows: standard culture medium without FBS (Serum-free); standard culture medium with 10 μM 5-fluorouracil (5-FU) (Sigma-Aldrich, MO, USA); standard culture medium pre-conditioned in hypoxia chamber for 48 hrs (Hypoxia). For all experiments, except those in hypoxic conditions, cells were incubated with the appropriate culture media in a humidified incubator at 37 °C with 5% CO_2_ for the duration of the experiment. For experiments in hypoxic conditions, cells were maintained inside a modular incubator chamber (Hillups-Rothenberg, CA, USA) in which humidity was maintained by addition of a 60 mm Petri dish containing 5 ml of water. Prior to each use, the chamber was flushed with 5% CO_2_, 1% O_2_, and N_2_ before being sealed and stored at 37 °C in an incubator. Cells were removed from the chamber for not more than one hour to conduct experiments, after which time the chamber was again flushed with 5% CO_2_, 1% O_2_, and N_2_ before being sealed and stored at 37 °C. Hypoxic conditions were confirmed using a Cyto-ID Hypoxia Detection Kit (Enzo Life Sciences, NY, USA) and fluorescence microscopy detection according to the manufacturer’s instructions.

### Amniotic fibroblasts: general information, culture conditions, and treatments

Passage 1–3 fibroblast cultures were established from surplus amniocentesis samples used in pre-natal diagnosis. Two cases of constitutional trisomy 13 (gestational age 16 and 22 weeks) and three diploid controls (gestational age 13, 15, and 16 weeks) were used in our study. FISH analysis of interphase nuclei stained with a chromosome 13 specific probe showed that the trisomic cell populations used in this study were highly homogenous (>95%) for the trisomic karyotype. The study acknowledged the ethics guidelines under Portugal national rules and according to the principles of the Declaration of Helsinki, and was approved by the Ethics Committee of Hospital de S. João-Porto (dispatch 14, Nov 2012). Informed consent forms with detailed information were provided to all patients. The study did not imply collection of extra material from the healthy female donors (only surplus cells/tissues were used); the study did not bring any direct benefits to the volunteers; there were no risks or costs for the volunteers; there was no access to patient clinical data (samples were obtained in anonymous form from the Hospital Genetics Department); participation was volunteer and could be interrupted at any time; there are no ethical impacts predicted; there will be no commercial interests. Amniotic fibroblasts were maintained in EMEM (Lonza, Switzerland) supplemented with 15% FBS, 2.5 mM glutamine, and 1x antibiotic-antimycotic solution (all from Gibco, NY, USA). All cells were kept in a humidified incubator at 37 °C with 5% CO_2_. For exposure to selective conditions, culture medium was prepared as follows: standard culture medium without FBS (serum-free); standard culture medium with 5 μM 5-fluorouracil (5-FU) (Sigma-Aldrich, MO, USA). A lower dose of 5-FU was used in AF compared to DLD1 cells due to the AF’s higher sensitivity to the drug.

### Growth curves

To determine the proliferative capacity of CRC cells over time, we plated each DLD1 cell line in a 6-well plate (6 wells total) at a density of 1.5 × 10^5^ cells per well. Following 24 hrs of growth in standard media, the cells were washed twice in PBS and re-incubated under selective culture conditions. For the 4-6-day samples, the media was replenished half-way through the experiment. At 24-hour intervals for six days, the media from one well of each DLD1 cell line was removed, the cells were washed twice with PBS and trypsinized. Cells were then vigorously re-suspended in media to a final total volume of 3 ml. From this, a 100 μl sample was thoroughly pipetted and mixed with an equal amount of 0.4% trypan blue solution (Gibco, NY, USA), and subjected to cell counting. Cell counts were performed by hemocytometer (Bright-Line, PA, USA) and averaged from four samples.

To determine the proliferative capacity of amniotic fibroblasts over time, we were unable to use the same method used in CRC cells due to technical difficulties. Instead, we used a different method, as described below. We plated each trisomy 13 and diploid control in 6-well plates (7 wells total) at a density of 0.8–1 × 10^5^ cells per well. Following 24 hrs of growth in standard media, the cells were rinsed in PBS and re-incubated under selective culture conditions. One well was immediately fixed with cold methanol and stained with DAPI for nuclei quantification (day 0). The other six wells were fixed in methanol and DAPI-stained at 24-hour intervals over a six-day period (days 1–6). To quantify the number of cells, images of stained nuclei in a total of 100 neighboring fields (covering a 1 cm^2^ area) at two different locations in each well were acquired with a 10× objective on a Leica DMI 6000B motorized inverted epi-fluorescence microscope. The results from the two different stitched fields were averaged representing the number of nuclei per cm^2^. This number was then extrapolated to the total area of the well (9.6 cm^2^). The growth curves represent the mean ± S.D. values calculated from independent experiments using different trisomic and diploid samples.

### Mitotic index analysis

1.5 × 10^5^ CRC cells suspended in standard media were plated on 22 × 22 mm acid-washed sterile glass coverslips inside 35 mm Petri dishes. After 24 hours, cells were incubated under the appropriate culture conditions for additional 24 hrs. Cells were then washed twice with PBS, fixed with freshly prepared 4% paraformaldehyde (Fischer Scientific, NJ, USA), washed twice more with PBS, and DAPI stained. For each sample, the number of mitotic cells was counted over a total of at least 1,000 cells.

### TUNEL assay

0.5 × 10^5^ cells suspended in standard media were plated on 22 × 22 mm acid-washed sterile glass coverslips inside 35 mm Petri dishes. After 24 hours, cells were incubated under the appropriate culture conditions for additional 24 hrs. Cells were then washed twice with PBS, fixed with freshly prepared 4% paraformaldehyde (Fischer Scientific, NJ, USA), washed twice more with PBS + 0.01% sodium citrate, and finally stained using an *in situ* cell death detection kit (Roche, Basel, Switzerland). Cells were then washed three times with PBS before DAPI staining. For each sample, the number of TUNEL positive cells was recorded over a total of at least 1,000 cells. Positive and negative controls were used according to the manufacturer’s guidelines.

### Fluorescence microscopy

For both mitotic index and TUNEL assay quantification, samples were viewed on a Nikon Eclipse TE2000-U inverted microscope (Nikon Instruments Inc., NY, USA) equipped with a swept field confocal system (Prairie Technologies, WI, USA), a 100×/1.4 NA Plan-Apochromatic objective, and an automated ProScan stage (Prior Scientific, Cambridge, UK). The confocal head was accessorized with a multiband pass filter set for illumination at 405, 488, 561, and 640 nm and illumination was obtained through an Agilent MLC400 monolithic laser combiner (Agilent Technologies, CA, USA) controlled by a four channel acousto-optic tunable filter. Digital images were acquired with a HQ2 CCD camera (Photometrics, AZ, USA). Stacks of images were acquired through the Z axis at 0.6 μm steps. Exposure time, Z-axis position, laser line power, and confocal system were all controlled by NIS Elements AR software (Nikon Instruments Inc., NY, USA) on a PC computer (Dell, TX, USA).

### Soft agar assay

1.5 ml of a 1:1 mixture of 2% agar (Fischer Scientific, NJ, USA) and 2× DMEM (Gibco, NY, USA) media (prepared according to the specific selective condition for each sample) was poured evenly into a 35 mm Petri dish. After cooling, 1.5 ml of a 1:1 mixture of 1.4% agar (Fischer Scientific, NJ, USA) and 2× DMEM (prepared according to the specific culture condition for each sample) media was used to re-suspend 1 × 10^5^ cells and then poured on the bottom solidified agar layer. After solidification of the top agar layer, 2 ml of RPMI 1640 media (prepared according to the specific selective condition for each sample) were added and the samples incubated in a humidified incubator at 37 °C with 5% CO_2_, or in the hypoxia chamber. The colonies were grown for 22 days and media changed every 4–5 days. Ten randomly selected fields of view were imaged on a Nikon Eclipse Ti inverted microscope (Nikon Instruments Inc., NY, USA) equipped with a 20×/0.4 NA ADL phase contrast objective, phase-contrast transillumination, transmitted light shutter, ProScan automated stage (Prior Scientific, MA, USA), and a HQ2 CCD camera (Photometrics, AZ, USA). For each field of view, Z-stacks were acquired by imaging 10 focal planes at 100 μm steps and colony number and size were measured for colonies present on different focal planes. The total number of colonies was quantified for all ten fields of view and the size of each colony was quantified by measuring the length of the longest axis. hTERT-RPE1 cells were used as a negative control and as a baseline for colony size measurements. Individual dot-like structures, corresponding to 1-few non-proliferating cells, were observed at low density in hTERT-RPE1 samples. The average size of these structures was 15 μm, and this was considered as the lower size limit for colony size measurements in DLD1 cell lines.

### Matrigel invasion assay

The matrigel invasion assay was performed using an 8 μm pore transwell PET membrane (BD Biosciences Inc., MA, USA). The matrigel mixture (Corning Inc., MA, USA) was reconstituted with the appropriate culture media to a concentration of 200 μg/ml and poured evenly over the transwell PET membrane; 10^5^ cells were re-suspended in RPMI 1640 media and plated onto the matrigel; 500 μl of media were also added to the bottom well and the chambers were incubated. After 24 hrs, both the media in the bottom well and that in the top chamber were replaced with media prepared according to the specific culture condition for each sample. After incubating for additional 24 hrs, non-invasive cells were scraped off the chamber side of the transwell membrane and invasive cells were fixed with 100% methanol, washed twice with PBS, and stained with a 1% Giemsa solution. Quantification was performed by light microscopy on a Nikon Eclipse TS100, using a 20×/0.4 NA ADL phase contrast objective and counting the total number of cells from 6 random fields of view.

## Additional Information

**How to cite this article**: Rutledge, S. D. *et al.* Selective advantage of trisomic human cells cultured in non-standard conditions. *Sci. Rep.* **6**, 22828; doi: 10.1038/srep22828 (2016).

## Supplementary Material

Supplementary Information
