# Author Correction: Selective advantage of trisomic human cells cultured in non-standard conditions

**DOI:** 10.1038/s41598-022-19297-z

**Published:** 2022-08-31

**Authors:** Samuel D. Rutledge, Temple A. Douglas, Joshua M. Nicholson, Maria Vila-Casadesús, Courtney L. Kantzler, Darawalee Wangsa, Monika Barroso-Vilares, Shiv D. Kale, Elsa Logarinho, Daniela Cimini

**Affiliations:** 1 Department of Biological Sciences, Blacksburg, VA 24061 USA; 2 Biocomplexity Institute, Virginia Tech, 1015 Life Sciences Circle, Blacksburg, VA 24061 USA; 3 Biomedical Engineering, Virginia Tech, Blacksburg, VA 24061 USA; 4 Bioinformatics Platform, CIBERehd, Barcelona, Spain; 5 Genetics Branch, National Cancer Institute, NIH, Bethesda, MD 20892 USA; 6 Aging and Aneuploidy Laboratory, Instituto de Biologia Molecular e Celular, Instituto de Investigação e Inovação em Saúde – i3S, Universidade Do Porto, Rua Alfredo Allen 208, 4200-135 Porto, Portugal; 7 Cell Division Unit, Department of Experimental Biology, Faculdade de Medicina, Universidade do Porto, Alameda Prof. Hernâni Monteiro, 4200-319 Porto, Portugal

Correction to: *Scientific Reports* 10.1038/srep22828, published online 09 March 2016

This Article contains an error in Figure 4A, where the panel ‘DLD1+7’ was a duplicate of the panel shown in Figure 4C ‘DLD1+7’. Additionally, the duplicated panel was incorrectly rotated by 90 degrees. The correct Figure 4 and accompanying legend appear below.

**Figure 4:**
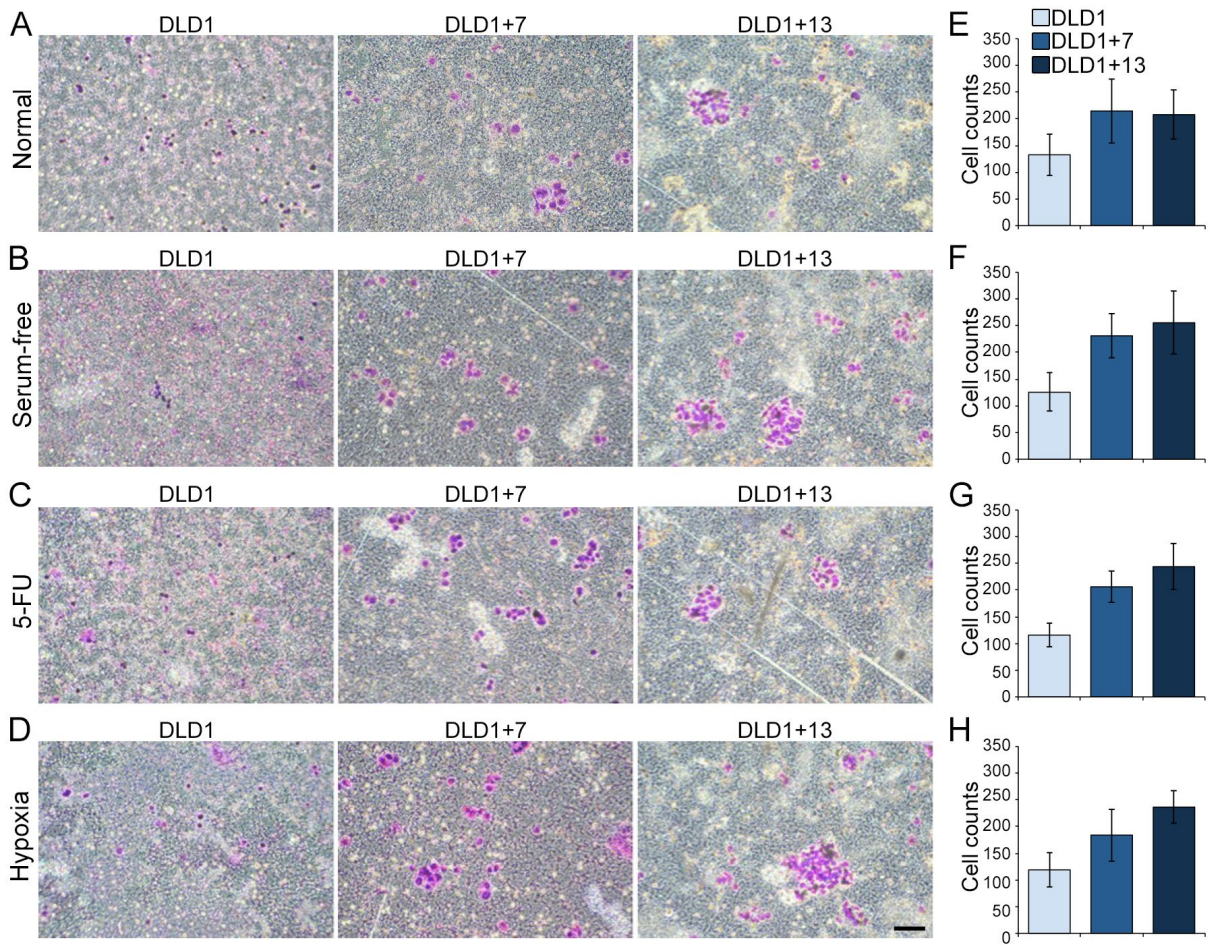
Aneuploidy increases invasiveness of CRC cells. The invasive capacity of the three different cell lines was assessed using a matrigel invasion assay. (**A–D**) Examples of Giemsa-stained invasive DLD1, DLD1+7 and DLD1+13 cells cultured under different conditions. Scale bar, 100 μm. (**E–H**) Quantification of invasive DLD1, DLD1+7 and DLD1+13 cells cultured under different conditions. The data are reported as mean and s.e.m. from three biological replicates. Statistical analysis showed that significantly larger numbers of aneuploid compared to diploid cells migrated through the matrigel layer (t-test, p < 10^−4^ for each aneuploid CRC cell line compared to diploid cells under all culture conditions).

