# Supplementary material for: Selective advantage of trisomic human cells cultured in non-standard conditions
Source: Sci Rep. 2016 Mar 9;6:22828. doi: 10.1038/srep22828 (PMC4783771; doi:10.1038/srep22828)
Supplement: Supplementary Information [file srep22828-s1.pdf]

Supplementary Information

Selective advantage of trisomic human cells cultured in non-standard conditions.

Samuel D. Rutledge[1,2], Temple A. Douglas[2,3], Joshua M. Nicholson[1,2], Maria Vila-Casadesús[4], Courtney L. Kantzler[1,2], Darawalee Wangsa[5], Monika Barroso-Vilares[6], Shiv D. Kale[2], Elsa Logarinho[6,7], Daniela Cimini[1,2]*

[1]Department of Biological Sciences, [2]Virginia Biocomplexity Institute, Virginia Tech, 1015 Life Sciences Circle, Blacksburg, VA 24061 – USA
[3]Biomedical Engineering, Virginia Tech, Blacksburg, VA 24061 – USA
[4]Bioinformatics Platform, CIBERehd, Madrid, Spain
[5]Genetics Branch, National Cancer Institute, NIH, Bethesda, MD, 20892 – USA
[6]Aging and Aneuploidy Laboratory, Instituto de Biologia Molecular e Celular, Instituto de Investigação e Inovação em Saúde – i3S, Universidade do Porto, Rua Alfredo Allen 208, 4200-135 Porto – Portugal
[7]Cell Division Unit, Department of Experimental Biology, Faculdade de Medicina, Universidade do Porto, Alameda Prof. Hernâni Monteiro, 4200-319 Porto – Portugal

*Corresponding author:
Daniela Cimini
Department of Biological Sciences & VBI
1015 Life Science Circle
Blacksburg, VA 24061
USA
e-mail: cimini@vt.edu

Mathematical model, parameters, and assumptions.

To determine whether the experimentally observed mitotic indices and rates of apoptosis (Figures 2B, F) could explain the growth trends (Figure 1) of the three DLD1 CRC cell lines cultured under different conditions, we used a deterministic mathematical model, in which we assumed the growth to be exponential and be described by the following equation:

$$\frac{dN}{dt}(\alpha - \beta)N \qquad (1)$$

In this model, N represents the number of cells, t is time, α is the rate of cell division, and β is the rate of apoptotic cell death.
The solution to this differential equation is the exponential growth equation, shown below:

$$N = N_0 e^{(\alpha-\beta)t} \qquad (2)$$

The use of such a deterministic model is justified because we can assume that (i) our cells are not approaching carrying capacity; (ii) culture conditions are maintained constant; (iii) there is no competition from other cell types (as could be the case in vivo).

In order to determine the rates of cell division and apoptotic death, we needed to estimate the times needed for cell division and apoptotic death (TUNEL-positive state) to occur. Cell division times were experimentally measured for each of the three cell lines (DLD1, DLD1+7 and DLD1+13) using phase contrast live-cell microscopy and measuring the elapsed time from cell round-up to anaphase (Table S1).

Table S1. Duration of mitosis in the three CRC cell lines.

Cell line	Duration of mitosis (mean ± S.D.)	N
DLD1	34 ± 7 minutes	131
DLD1+7	38 ± 12 minutes	107
DLD1+13	28 ± 10 minutes	212

As for the time required for apoptotic cell death, although we knew the frequencies of TUNEL-positive cells in the population (Figure 2F), it is not known how long cells persist in a TUNEL-positive state. Therefore, we needed to estimate this time. We tested a range of times, but found that TUNEL-positivity times above 60 minutes did not significantly affect the theoretical results, whereas times significantly below 45 minutes resulted in very low rates of theoretical growth (i.e., very high rates of cell death), which is inconsistent with our experimental data. Moreover, given that the overall apoptotic process is known to take hours to be completed[1], it is reasonable to believe that the stage during which cells persist as TUNEL-positive is unlikely to be shorter than 45-60 minutes. Thus, the data presented here consist of two sets, in which TUNEL-positivity time was either 45 or 60 minutes.

From the cell division and TUNEL-positivity values, we calculated the following as rates of cell division and apoptotic cell death:

$$\alpha = \frac{mitotic\ index}{time\ in\ mitosis}\ ; \quad \beta = \frac{fraction\ of\ TUNEL{-}positive\ cells}{time\ cells\ persist\ as\ TUNEL{-}positive}$$

The values for mitotic indices and fraction of TUNEL-positive cells for each CRC cell line under different culture conditions were obtained from the data reported in Figure 2 and are summarized in Table S2.

Table S2. Initial population size (from data reported in Figure 1), mitotic indices, and fractions of TUNEL-positive cells for each CRC cell line under different culture conditions.

Standard

Cell line	Initial cell count	Mitotic Index (mean ± S.D.)	Fraction of TUNEL-positive cells
DLD1	212,917	0.058645104 ± 0.006862652	0.001175963
DLD1+7	117,917	0.050974638 ± 0.003358715	0.004092463
DLD1+13	120,417	0.042177766 ± 0.002223556	0.014365527

Serum-free

Cell line	Initial cell count	Mitotic Index (mean ± S.D.)	Fraction of TUNEL-positive cells
DLD1	132,083	0.031570189 ± 0.006544618	0.014623089
DLD1+7	185,833	0.044841685 ± 0.007695407	0.004069802
DLD1+13	188,750	0.041570878 ± 0.005468283	0.008688263

5-FU

Cell line	Initial cell count	Mitotic Index (mean ± S.D.)	Fraction of TUNEL-positive cells
DLD1	87,083	0.034740967 ± 0.005380910	0.034234035
DLD1+7	185,000	0.042105044 ± 0.008053757	0.028640811
DLD1+13	227,083	0.041325575 ± 0.005077052	0.017294774

Hypoxia

Cell line	Initial cell count	Mitotic Index (mean ± S.D.)	Fraction of TUNEL-positive cells
DLD1	94,583	0.055779498 ± 0.007105617	0.019471951
DLD1+7	71,667	0.047794669 ± 0.002031374	0.031187891
DLD1+13	112,083	0.057347095 ± 0.004674209	0.013723979

Comparison of theoretical and experimental data.

Using the exponential growth equation (2) and the parameters and assumptions described in the previous section, we calculated theoretical growth curves (Figures S1 and S2).

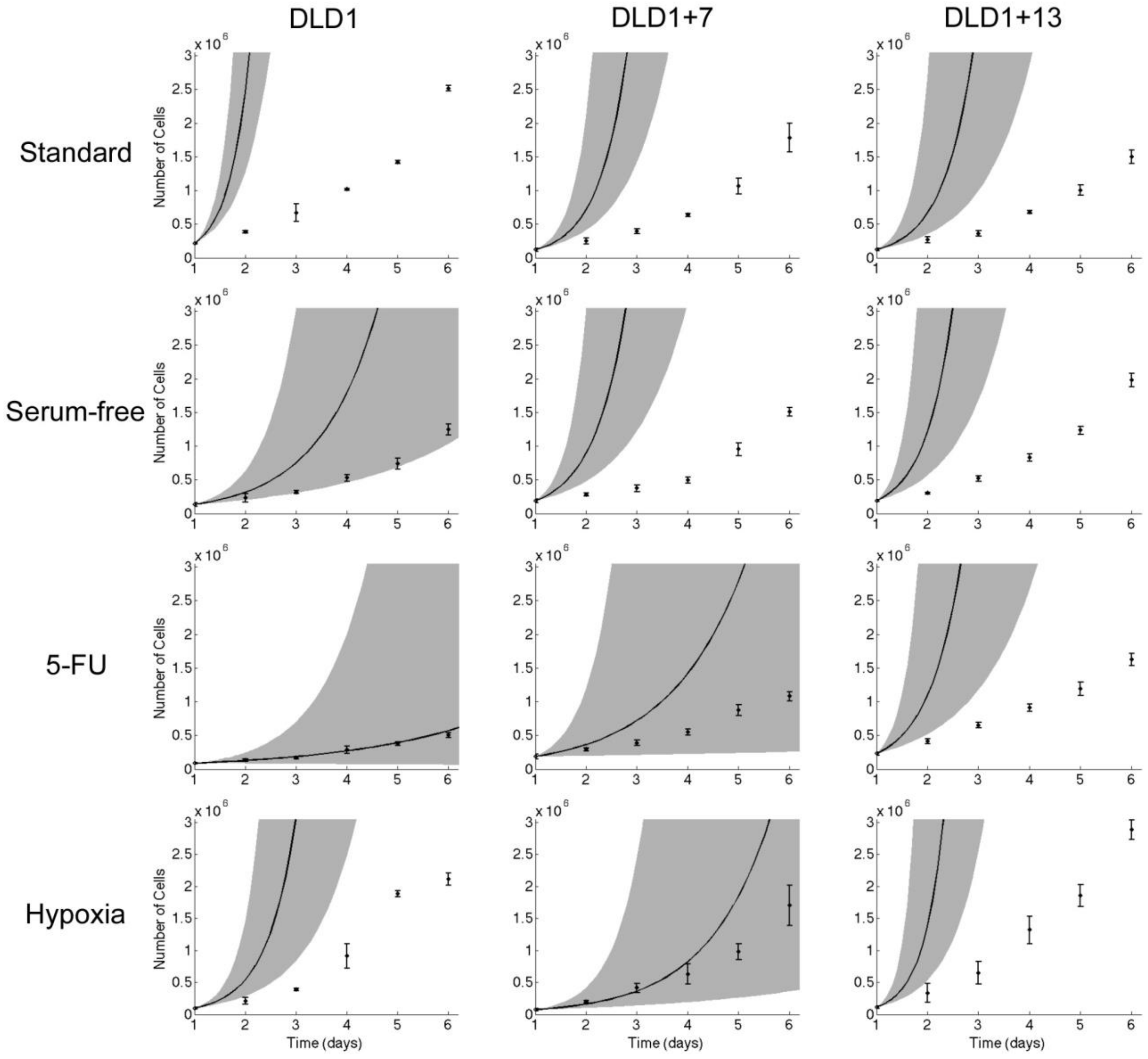

Figure S1. Plots show theoretical and experimental growth curves. Points with error bars are experimental results obtained by cell counts (as in Figure 1). Solid black lines show theoretical growth curves given experimentally defined mitotic indices (Table S2), mitotic timing (Table S1), and TUNEL-positivity values (Table S2) and with a set TUNEL-positivity duration of 45 minutes. Grey regions show variance in theoretical values based on variance in mitotic indices and mitotic duration.

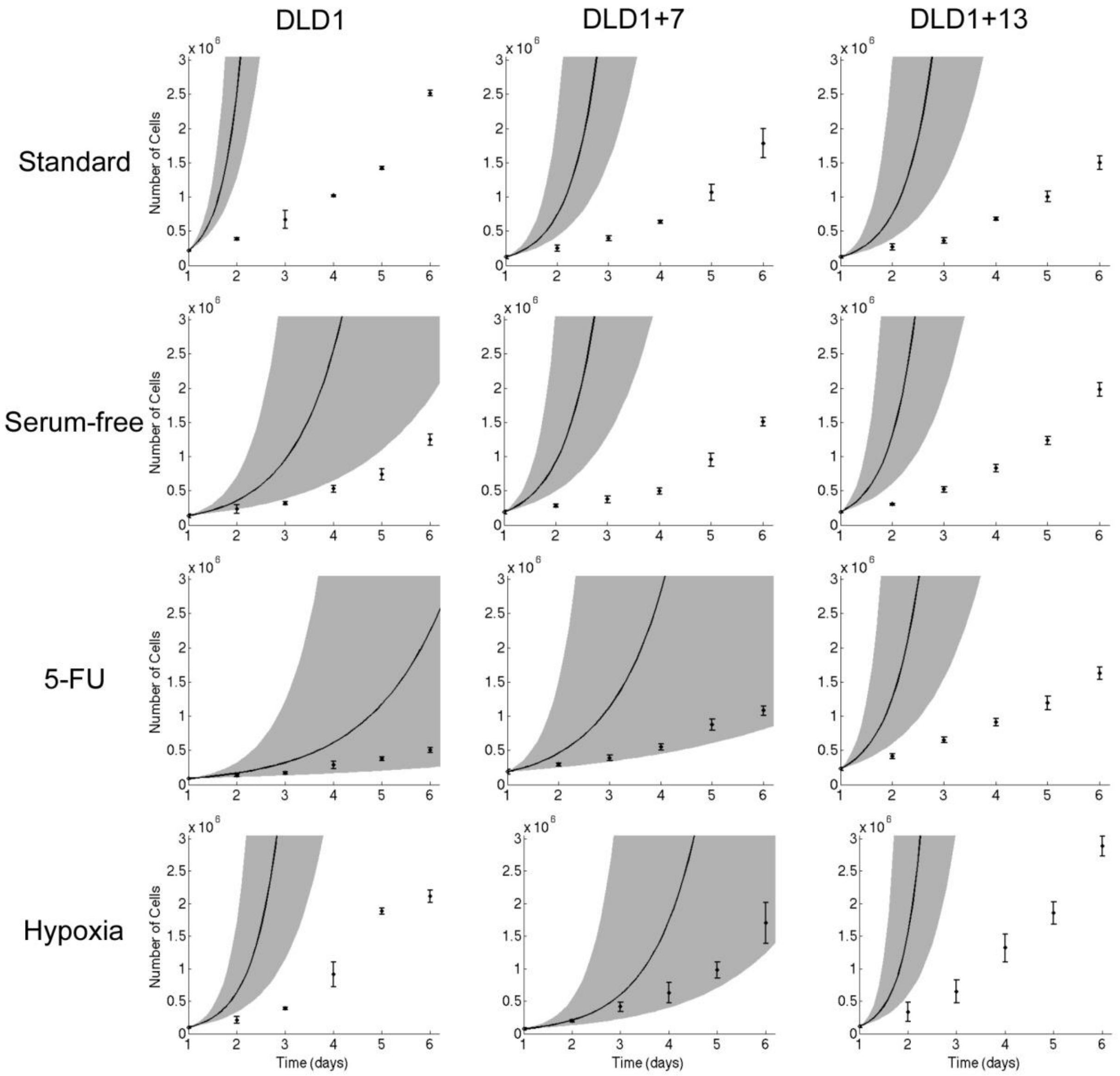

Figure S2. Plots show theoretical and experimental growth curves. Points with error bars are experimental results obtained by cell counts (as in Figure 1). Solid black lines show theoretical growth curves given experimentally defined mitotic indices (Table S2), mitotic timing (Table S1), and TUNEL-positivity values (Table S2) and with a set TUNEL-positivity duration of 60 minutes. Grey regions show variance in theoretical values based on variance in mitotic indices and mitotic duration.

The theoretical data did not closely match the experimental data, generally showing faster growth compared to the experimental curves. Thus, we set out to estimate the difference between the two data sets. The experimental growth curve can be represented as:

$$\frac{dN}{dt} = kN \tag{3}$$

where k represents the change in the population, including birth of new cells due to mitosis and death due to any cause. We can rewrite our theoretical growth curve as:

$$\frac{dN}{dt} = (\alpha - \beta + \gamma)N \tag{4}$$

In this curve, all values are the same as in equation (1), but the term γ is added to account for contributing factors that were not accounted for by the growth rate or the death rate in equation (1). By curve fitting to the experimental data, we can extrapolate k, and then find γ as the difference between the experimental growth curve and the theoretical growth curve. Thus, we first obtained k values for different CRC cell lines under different culture conditions by fitting the curve described by equation (3) to the experimental data set. The k values obtained in such way are reported in Table S3.

Table S3. Experimental k values.

**Standard**		
**Cell line**	***k***	**R^2**
DLD1	0.4724	0.99502
DLD1+7	0.4559	0.95762
DLD1+13	0.4308	0.95641

**Serum Free**		
**Cell line**	***k***	**R^2**
DLD1	0.3794	0.96156
DLD1+7	0.3841	0.97646
DLD1+13	0.4467	0.99417

**5-FU**		
**Cell line**	***k***	**R^2**
DLD1	0.2622	0.81579
DLD1+7	0.3452	0.99362
DLD1+13	0.4019	0.98202

**Hypoxia**[#]		
**Cell line**	***k***	**R^2**
DLD1	0.5494	0.92081
DLD1+7	0.4892	0.89330
DLD1+13	0.5664	0.95077

[#]Note, the k values for all cell lines under hypoxia are higher than the k values under standard conditions, confirming the higher proliferation rates of all three cell lines in hypoxia compared to their proliferation in standard culture conditions.

Next, by subtracting $(\alpha - \beta)$ from these k values, we determined γ values for different CRC cell lines under different culture conditions. These γ values are reported in Table S4.

Table S4. γ values.

Standard

Cell line	γ (45 min; 60 min)
DLD1	-1.9693; -1.9787
DLD1+7	-1.2746; -1.3074
DLD1+13	-1.2185; -1.3335

Serum Free

Cell line	γ (45 min; 60 min)
DLD1	-0.4339; -0.5508
DLD1+7	-1.1570; -1.1896
DLD1+13	-1.3898; -1.4593

5-FU

Cell line	γ (45 min; 60 min)
DLD1	-0.0197; -0.2936
DLD1+7	-0.3227; -0.5518
DLD1+13	-1.1904; -1.3287

Hypoxia

Cell line	γ (45 min; 60 min)
DLD1	-1.0830; -1.2388
DLD1+7	-0.2095; -0.4590
DLD1+13	-1.8782; -1.9880

All the γ values were negative, indicating that in all cases the experimental growth was lower than the theoretically predicted growth, as also evident in Figures S1 and S2. All the parameters used for the simulations were experimentally measured, except the TUNEL-positivity time. If the TUNEL-positivity time parameters were the only contributing factor to the differences between theoretical and experimental growth curves, we would expect the see uniformity in the γ values. The fact that the γ values were different under different culture conditions, suggests that in our experiments either cell proliferation occurred at lower rates than calculated based on mitotic indices and mitotic duration or that cells were dying more than what the TUNEL assay data indicated. Lower cell proliferation could be caused by cell cycle delays. However, this would be reflected in the rates of mitosis, given that arrest/delay in a cell cycle stage other than mitosis would result in fewer cells entering mitosis. Therefore, the mitotic parameters used for the model already account for such possible cell cycle delay(s). Instead, our only measure of cell death was based on the TUNEL assay, which may allow for detection of some forms of cell death, but not others[2]. Thus, we suggest that the differences

between the experimental and theoretical data can be explained by cell death occurring at slightly higher rates than those estimated via TUNEL assay.

References

1 Saraste, A. & Pulkki, K. Morphologic and biochemical hallmarks of apoptosis. *Cardiovasc Res* **45**, 528-537 (2000).

2 Sperandio, S., de Belle, I. & Bredesen, D. E. An alternative, nonapoptotic form of programmed cell death. *Proc Natl Acad Sci U S A* **97**, 14376-14381, doi:10.1073/pnas.97.26.14376 (2000).
